# Commentary: Intermittent Hypoxia Severity in Animal Models of Sleep Apnea

**DOI:** 10.3389/fphys.2019.00609

**Published:** 2019-05-22

**Authors:** Jonathan C. Jun, Erik R. Swenson

**Affiliations:** ^1^Pulmonary and Critical Care Medicine, School of Medicine, Johns Hopkins University, Baltimore, MD, United States; ^2^Pulmonary and Critical Care Medicine, University of Washington, Seattle, WA, United States

**Keywords:** hypoxia, hemoglobin, dissociation, sleep apnea, altitude

Obstructive sleep apnea (OSA) is a common disorder that leads to problems including intermittent hypoxia (IH) and arousals from sleep. To simulate consequences of OSA, some studies expose rodents to IH with the intention of simulating the oxygen profile experienced by OSA patients. Some IH experiments induce hemoglobin oxygen saturation (SaO_2_) to fall to 50–70% during the nadir phase. In a recent review, we stated that this degree of hypoxemia is more severe than that experienced by typical OSA patients (Chopra et al., 2015). Farre et al. challenged this statement (Farré et al., 2018), pointing out that small mammals such as rodents have a right-shifted oxyhemoglobin dissociation curve (ODC) compared to humans (Schmidt-Nielsen, 1970). They argue that the higher arterial partial oxygen pressure (PaO_2_) for a given SaO_2_ confers mice with “better oxygen reserve.” To achieve PaO_2_ nadir values similar to those experienced by severe OSA patients they contend, “SaO_2_ in mice should be much lower than the SaO_2_ observed in patients.” We disagree with this statement, which relies on PaO_2_ as an indicator of tissue oxygenation. Arterial O_2_ content (CaO_2_) is determined by classic formula:

$$\begin{array}{l} \text{Ca}{\text{O}}_{2}=\left(1.34\text{ x Hb x Sa}{\text{O}}_{2}\right)+\left(0.003\text{ xPa}{\text{O}}_{2}\right) \end{array}$$

Systemic O_2_ delivery (DO_2_) is the product of blood flow (Q) and CaO_2_. From these equations, it is apparent that PaO_2_ as it determines the amount of dissolved O_2_ gas, itself has a negligible contribution to CaO_2_ or DO_2_. As oxygenated blood reaches target tissues, O_2_ dissociates from hemoglobin to maintain a favorable capillary-to-tissue PO_2_ driving gradient. When O_2_ diffuses into tissues, systemic capillary PaO_2_ falls, and is replenished by upstream oxygenated hemoglobin. Aerobic metabolic processes then consume cell O_2_. The balance between O_2_ supply and demand determines the cellular PO_2_. Therefore, cellular PO_2_–the parameter that truly determines oxygen adequacy - depends upon DO_2_, capillary density, and rates of O_2_ utilization. A right-shifted ODC means that O_2_ is unloaded from hemoglobin more rapidly, but this does not increase the total amount of O_2_ delivered.

To propose that mice should have their SaO_2_ lowered in order to achieve a PaO_2_ equal to that of humans is tantamount to suggesting that one should load a truck to only half its capacity, because it unloads boxes twice as quickly. If we invoke this logic, and use the figure provided by Farre et al., mouse SaO_2_ would have to be decreased to ~75% to match a normoxic human PaO_2_ at ~65 mm Hg. Mice exposed to hypoxemia of this magnitude exhibit robust erythrocytosis (Fagan, 2001) and activate anaerobic metabolic pathways (Jun et al., 2012, 2014) indicating that mice are effectively hypoxic at a higher PaO_2_ than humans. Their higher PaO_2_ may be necessary to maintain adequate tissue PO_2_ (as opposed to having “better oxygen reserve”), as mice have dramatically higher mass-specific metabolic rates than humans. Therefore, we should be targeting equivalent drops in SaO_2_ between humans and rodents if the goal is to reduce distal O_2_ delivery to the same extent.

Implicit in the argument by Farre et al. is that a right-shifted ODC is advantageous in mitigating effects of low SaO_2_. There is no evidence that we could find to support this argument. A right-shifted ODC is advantageous in states such as shock or hemorrhage ensuring O_2_ is maximally transferred to ischemic tissues (Agostoni et al., 1975; da Luz et al., 1975; Cornum et al., 1998; Morgan, 1999). Conversely, transfusion of blood depleted of 2,3 diphosphoglycerate to left-shift the ODC (Riggs et al., 1973) decreases tissue oxygen supply. These examples pertain to conditions when hemoglobin is fully O_2_ saturated (i.e., SaO_2_ is constant). What is the effect of shifting the ODC during hypoxemia (e.g., high altitude, OSA)? Here, effects of hemoglobin O_2_ affinity are not straightforward. Left shifting the ODC increases O_2_ uptake in the pulmonary capillaries, but compromises peripheral tissue O_2_ unloading. At high altitude, this trade-off is advantageous for survival; the “tipping point” occurs when O_2_ uptake becomes diffusion limited (Storz and Moriyama, 2008). Indeed, rats exposed to severe hypoxia survived longer with a left-shifted ODC (Eaton et al., 1974). The rightward ODC curve of rodents may actually be counter-productive in the setting of ambient hypoxia.

In conclusion, we should not “titrate” SaO_2_ in different species to match their PaO_2_, based on different hemoglobin O_2_ affinities. We stand by our statement that IH experiments that lower the SaO_2_ of mice to nadirs of 50–70% are severe. Our intent was not to dismiss the importance or validity of these IH models. We merely object to the claim that SaO_2_ needs to be lowered more in mice than humans to simulate consequences of OSA.

## Author Contributions

JJ and ES collaboratively wrote the manuscript.

### Conflict of Interest Statement

The authors declare that the research was conducted in the absence of any commercial or financial relationships that could be construed as a potential conflict of interest.
